# Vitamin D status and its correlates in Saudi male population

**DOI:** 10.1186/s12889-019-6527-5

**Published:** 2019-02-20

**Authors:** Karim H. Farhat, Mostafa A. Arafa, Danny M. Rabah, Hussein S. Amin, Nahla K. Ibrahim

**Affiliations:** 1 Cancer Research Chair, College of Medicine, King Saud University, P.O.Box: 76047, Riyadh, 11922 Saudi Arabia; 2 Laboratory of Molecular Immuno-Oncology, Faculty of Medicine of Monastir, Monastir, Tunisia; 3 Surgery Department, College of Medicine, King Saud University, Riyadh, Saudi Arabia; 4 Family and Community Medicine Department College of Medicine, King Saud University, Riyadh, Saudi Arabia; 5 Epidemiology Department High Institute of Public health, Alexandria University, Alexandria, Egypt

**Keywords:** Vitamin D levels, Saudi male, Vitamin-D deficiency, 25-Hydroxyvitamin D

## Abstract

**Background:**

Vitamin-D deficiency is a universal health problem caused primarily by inadequate exposure to sunlight. This study aimed to assess the vitamin D status and investigate the factors affecting vitamin D distribution among Saudi males.

**Methods:**

A cross-sectional study was conducted at the King Saud University Medical City from December 2015 to August 2016. Saudi males attending the outpatient primary health care clinics were invited to participate in the study. Data were collected on the current and past health status along with biochemical investigations for total 25-hydroxyvitamin D (25OHD), blood sugar, and cholesterol.

**Results:**

Majority of the participants (76.1%) had vitamin D deficiency. Blood sugar level, age, and cholesterol level were the most significant factors associated with vitamin D status. The highest percentage of deficiency was observed in the youngest age group (30-40 years). With increasing age, the percentage of deficiency decreased significantly. Those with normal blood sugar and cholesterol level had higher serum vitamin D levels compared to those with diabetes and hypercholesterolemia.

**Conclusions:**

Vitamin D deficiency is still endemic in Saudi Arabia, particularly among younger males and those with diabetes and hypercholesterolemia. Vitamin D screening, supplementations, and vitamin D-fortified foods should be provided especially for these groups.

**Electronic supplementary material:**

The online version of this article (10.1186/s12889-019-6527-5) contains supplementary material, which is available to authorized users.

## Background

Vitamin D deficiency is a universal health problem caused primarily by inadequate exposure to sunlight. Vitamin D is an important vitamin with powerful effects on several systems of the body [1]. Unlike most vitamins, vitamin D functions as a hormone, and every cell in the body has a receptor for it. The main source of vitamin D is sunlight in addition to certain types of foods such as fatty fish and fortified dairy products [2]. Nearly 1 billion people are suffering from vitamin D deficiency or insufficiency worldwide, and it is especially high among the elderly population [3], girls, and women from the Middle East [4]. Risk factors that are commonly associated with vitamin D deficiency include darker skin color, staying indoors, being overweight or obese, being elderly, low intake of fish or milk and milk products, and kidney or liver diseases [2].

A severe vitamin D deficiency could lead to variety of presentations like fatigue, body aches, and myopathy up to association with increased risk of prostate cancer, dementia, Schizophrenia and cardiovascular disease [2, 5]. Saudi Arabia that has a sunny environment throughout the year with very high temperatures especially in summer, one would expect very low vitamin D deficiency. However, due to the traditional clothing style of Saudi men that covers almost the whole body, in addition to mostly indoor activities and their routine during the day, they are inadequately exposed to sunlight. Hence, vitamin D deficiency is a common health issue in Saudi adults, particularly among females and in younger age groups; however, its reported prevalence varies between studies. The prevalence of deficiency has been estimated between 28-75% [6–8]. In addition, the lack of uniform cutoffs used to define deficient and sufficient serum levels of vitamin D makes it difficult to compare the prevalence rate of vitamin D deficiency between studies.

The current study was carried out to assess the status of vitamin D levels among Saudi males and investigate the significant correlations that influence the vitamin D levels in this cohort.

## Methods

This cross-sectional study was conducted at the King Saud University Medical City from December 2015 to August 2016. All Saudi males attending the outpatient primary health care clinics during the morning shift were invited to participate in the study. Written informed consent was obtained from all the participants before commencement of the study. A short questionnaire was administered to collect data on the current and past health status, history of smoking, prostate diseases, vitamin D supplementation, and sunlight exposure (Additional file 1). Anthropometric parameters such as height, weight, along with their blood pressure were also measured. Body Mass Index (BMI) was calculated.

Biochemical investigations included analyzing blood samples for total 25-hydroxyvitamin D (25OHD), sugar level, and lipid profile. Blood samples (4 ml) were collected from all participants in the morning and serum samples were isolated. The sera were centrifuged at 2000 G for 10 mins and stored at -20^o^ C until further analysis. The samples were analyzed for total 25OHD levels by the T-Vitamin D kit (Total vitamin D) using the Roche diagnostics test.

Age was categorized into 4 groups: 30-40 years, 41–50 years, 51–60 years, and above 60 years. According to World Health Organization (WHO) [9] classification, BMI was categorized as: underweight, BMI < 18.5 kg/m^2^; normal, BMI 18. 5-24.9 kg/m^2^; overweight, BMI 25. 5-29.9 kg/m^2;^ and obese, BMI ≥30 kg/m^2^.

According to the laboratory of King Saud University Medical City reference values, vitamin D deficiency was defined based on serum levels of 25OHD: low, 25OHD < 75 nmol/L; low normal to high normal, 25OHD between 75 and 250 nmol/L; and toxic, 25OHD > 250 nmol/L.

According to the National Health and Nutrition Examination Survey (NHANES), diabetes mellitus (DM) patients were identified as those participants with measured Hemoglobin A1c (HbA1c) levels ≥6.5% or those taking medication for diabetes. HbA1c levels were categorized as: normal (< 5.7%) and pre-diabetic (5. 7-6.4%). Participants were identified as having hypercholesterolemia if the cholesterol level was ≥6.2 mmol/L.

It is worth mentioning here that all Saudi populations are related to the same ethnic group, there is no difference in skin color, and all males wear similar traditional clothing.

The exclusion criteria were participants with chronic conditions that affect vitamin D status such as malabsorption, chronic liver disease, renal impairment or nephrotic syndrome; those who were on medications that can affect vitamin Df metabolisms such as anticonvulsants or corticosteroids; and those with a family history of hypocalcemia or vitamin D disorders.

### Statistical analysis

The data were expressed as mean and standard deviation. One way analysis of variance (ANOVA) and t-test were used to test the significance of the difference in vitamin D levels between more than two groups and two groups respectively. Factors affecting vitamin D were determined using linear regression analysis. *P* values < 0.05 using a confidence interval (CI) of 95% were considered significant.

## Results

The total number of participants who met the inclusion criteria of the study was 1702. All of them were residents of Riyadh or the neighboring cities where they had plenty of sunlight exposure. The age ranged from 30-95 years and the mean age was 54.25 ± 13.1 years.

**Fig. 1:**
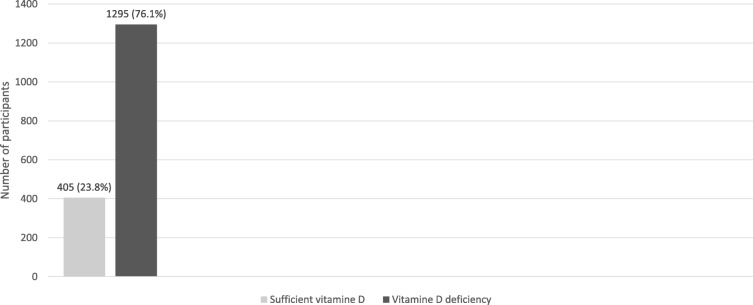
Distribution of vitamin D among Saudi males

Figure 1 illustrates that 1295 (76.1%) of the participants had vitamin D deficiency, while only 405 (23.8%) had normal or sufficient levels of vitamin D. Linear regression was used to identify the significant correlates of vitamin D status. The model included factors that might influence vitamin D status; i.e., age, BMI, blood sugar level, cholesterol level, hypertension, and vitamin D supplementation, and indicated that blood sugar level, age, and cholesterol level were the most significant correlates of vitamin D status among Saudi males (Table 1).

**Table 1:** Correlates of vitamin D distribution among studied Saudi males, linear regression analysis

Factor	β coefficient	t	Significance
Blood sugar level	0.103	3.6	.000
Age	0.07	2.4	.01
Blood cholesterol	0.06	2.1	.03
Hypertension	0.4	1.2	0.2
Vitamin D supplementation	0.2	0.89	0.7
BMI	0.05	1.7	.085

Exposure to sunlight varies between respondents; while 79% mentioned that they are exposed to sunlight daily, those who are exposed to sunlight, 3-4 times weekly and 1-2 time weekly represented 11.3 and 3.2% of our sample respectively, Those who are rarely exposed to sun comprised 6.5% of the participants. Only, more than one third (37.1%) of the participants mentioned that they spend more than one hour outside home exposing to sun, while the remaining percentage (62.9%) mentioned that their exposure to sunlight is barely less than one hour.

After excluding participants with toxic levels of vitamin D (> 250 nmol/L), Table 2 shows the distribution of mean serum vitamin D values across different age categories, blood sugar levels, and cholesterol levels. For the vitamin D deficient group (< 75 nmol/L), with a mean Vitamin D value of 36.91 ± 16.42 nmol/L, the highest percentage of deficiency was observed in the youngest age group of 30-40 years. Where the mean values of Vitamin D serum level was 36.91 ± 16.42 nmol/L, With increasing age, the vitamin D levels increased significantly with the lowest percentage of deficiency observed in the older age group of > 60 years. In the vitamin D sufficient group (75–250 nmol/L), the mean Vitamin D levels were lowest and the highest in the age groups of 50–60 years (97.5 ± 18.8 nmol/L) and > 60 years (104.72 ± 26.1 nmol/L) respectively. However, this difference was not statistically significant (F = 2.2, *p* = 0.08).

**Table 2:** Distribution of vitamin D serum level across age groups and blood sugar level

variables	Deficient vitamin D serum level < 75 nmol/L			Sufficient Vitamin D serum level 75-250 nmol/l		
	Mean + SD	F	*P* value	Mean + SD	F	*P* value
Age groups						
30–40 years	36.91 + 16.4			99.1 ± 25.8		
41–50 years	38.38 ± 16.4			103.2 ± 28		
51–60 years	40.51 ± 17.4			97.50 ± 18.8		
> 60 years	41.04 ± 18.8	3.5	0.01	104.72 ± 26	2.2	0.08
Blood sugar						
Not diabetic	41.3+ 18			101.9 ± 27.4		
Pre diabetic	39.1 ± 17	1.9	0.15	101.5 ± 21.1	1.1	0.3
Diabetic	39.1 ± 17.5			97.6 ± 22.5		
Cholesterol level						
Normal level	40.1 + 17.6	0.24	0.8	101.4 + 25.1	1.39	0.23
Hypercholesterolemia	39.6 + 17.1			96.7 + 17.9		

With regards to the blood sugar and cholesterol levels, the mean Vitamin D levels were higher in non-diabetic group and in those with normal cholesterol levels, for both groups (Vitamin D deficient and sufficient groups), though not statistically significant (*p* > 0.1) (Table 2). The mean values for 25OHD were significantly higher in non-diabetic group (56.03 ± 35.3 nmol/l) compared to the DM group (51.3 ± 30.2 nmol/l) (t = 2.6, *p* = 0.01). Similar results were seen in males with normal serum cholesterol levels vs. those with hypercholesterolemia, though not statistically significant (t = 1.49, *p* = 0.13) (Table 3). Most of the participants (73%) were not aware of their vitamin D levels at the start of the study.

**Table 3:** Comparison of the mean values of vitamin D across diabetic and hypercholesterolemia patients

Patients	Mean vitamin D level + SD	t	Significance
Diabetics	51.3 + 30.2	2.6	0.01
Non- diabetics	56.03 + 35.3		
hypercholesterolemia	49.8 + 27.9	1.49	0.13
normal cholesterol	55.42 + 33.6		

## Discussion

In the current study, 76.1% of the participants were found to be vitamin D deficient, which was higher than the figures reported in other studies. Age, blood sugar levels, and cholesterol were significant factors associated with vitamin D status in Saudi men.

Despite the higher serum 25OHD cut-off (75 nmol/L) used in the current study, the prevalence of vitamin D deficiency among this cohort of Saudi males was higher in comparison to other studies conducted in Saudi Arabia. Most of these studies have used a cut-off ranging from 25 nmol/L to 50 nmol/L, and the prevalence of vitamin D deficiency among Saudi Males ranged from 17.7 to 87.8% [10–13]. Using the most commonly used cut-off of 50 nmol/L, the prevalence of vitamin D deficiency in the present study would have been 52.1%. The higher prevalence of vitamin D deficiency reported in the current study highlights that though vitamin D deficiency in Saudi Arabia is still high, there is no evidence of any interventions to decrease these high figures. The higher prevalence of vitamin D deficiency may not be limited to developing countries as reported by Lenders et al., where vitamin D deficiency is common in as many as one half of the middle-aged to elderly subjects [14].

Age was one of the significant correlates of serum vitamin D levels in our study cohort. The vitamin D levels were lowest in the younger age group, which significantly increased until they reached the highest level in the older age group. These results are in agreement with earlier studies [10, 12, 15] conducted in Saudi Arabia and other countries, where young individuals are more likely to have insufficient levels of vitamin D, with the highest prevalence of vitamin D deficiency observed in the 20–30 years old age group [16]. On the other hand, Smotkin-Tangorra et al. [17] and Orwell et al. [18] concluded that higher prevalence of vitamin D deficiency was associated with older age and was more common in older males. It is a paradox, most of our respondents (79%) are exposed to sun daily; why younger males who are apparently healthy, have higher sunlight exposure, and engage in outdoor activities and exercises, have a higher prevalence of vitamin D deficiency. One explanation may be that older adults take supplements that contain vitamin D. However, this might not be the only reason and hence further large-scale studies to investigate this phenomenon are required.

With respect to the blood sugar levels, the mean vitamin D serum levels were higher amongst non-diabetic individuals in our study, which is in contrast to other studies that show higher levels of 25OHD in subjects with DM compared to non-diabetic individuals [19–23]. This may be attributed to medications that are used to treat DM which have been associated with enhancing circulating levels of 25OHD [24]. Earlier reports have shown that vitamin D may help regulate the production of insulin in the pancreas. It is supposed that body’s sensitivity to insulin is enhanced by vitamin D, which in turn minimizes the risk of insulin resistance that is often the precursor to diabetes type 2. Adjusting the levels of vitamin D in the blood to around 60–80 ng/ml can aid in maintaining the blood glucose levels under control, which is vital for diabetic patients [25]. Population studies suggested a positive correlation between low vitamin D levels with an increased possibility of developing type 2 diabetes. Hence, people with higher levels of vitamin D may have a low probability to develop type 2 diabetes [26].

Data on the association between cholesterol and vitamin D shows varied results. Population studies indicate that people with lower levels of vitamin D are more likely to have higher cholesterol levels. In 2012, a study showed that vitamin D supplements had no cholesterol lowering effects at least in the short term, and then too only low-density lipoproteins levels may increase. On the other hand, a study in 2014 found that taking calcium and vitamin D supplements together enhances cholesterol levels in postmenopausal overweight or obese women [27]. The study conducted in Pakistani Immigrants taking daily vitamin D supplementation of 10 or 20 μg for 1 year did not show any change in their lipid profile [28].

Our results indicated no significant association of BMI with vitamin D levels. Baradaran et al. reported results similar to our study [29], in contrast to earlier studies where vitamin D levels were found to be negatively correlated to BMI in both obese and non-obese population [30, 31].

### Study limitations

Though our study has some interesting findings, it has certain limitations. First, it was a cross-sectional, hospital-based study and therefore, we could not assess causality. In addition, this might have exposed the study to some sources of bias resulting from the manner in which study subjects were recruited or due to differences arising due to the participants’ cultural background, age, and socio-economic status. However, participants in the current study referred to the King Saud University Medical City were not limited to the capital city of Riyadh as all neighboring areas and governments centers refer to this tertiary hospital. This makes the selection of cases devoid of bias and could be considered representative of the Saudi population. Second, the sample size was relatively small and the dietary intake was not assessed. The study did not take into consideration the difference in sunlight exposure arising due to seasonal variation though, given the availability of sunshine nearly throughout the year, the seasonality might not be a significant factor in our case. However, the study addressed the significantly high deficiency of vitamin D amongst participants living in a country where they are exposed to sunny environment and high temperature throughout the year.

## Conclusion

Vitamin D deficiency is still endemic in Saudi Arabia, despite plenty of sun in the area. Increased awareness about the importance of vitamin D, particularly among younger age group, in addition to the integration of vitamin D testing in the primary health care centers, vitamin supplementations and vitamin D-fortified foods are warranted, especially for those with DM and hypercholesterolemia.

## Additional file

Additional file 1: Vitamin D distribution questionnaire. (PDF 235 kb)

## Abbreviations

25OHD25: hydroxyvitamin D
BMI: Body mass index
CI: Confidence interval
DM: Diabetes mellitus
HbA1c: Hemoglobin A1c
NHANES: National Health and Nutrition Examination Survey
T-VitD: Total vitamin D
WHO: World Health Organization
