# Supplementary material for: Vitamin D status and its correlates in Saudi male population
Source: BMC Public Health. 2019 Feb 20;19:211. doi: 10.1186/s12889-019-6527-5 (PMC6381691; doi:10.1186/s12889-019-6527-5)
Supplement: Supplementary file 1 — Vitamin D distribution questionnaire. (PDF 235 kb) [file 12889_2019_6527_MOESM1_ESM.pdf]

Vitamin D distribution among Saudi Men

Questionnaire

Hospital number: ………………………………….

Age: ……………Year

Are you a smoker ☐ Yes ☐ No

Do you have a prostate problem ☐ Yes ☐ No

If Yes, please verify

Are you getting vitamin D supplementation ☐Yes ☐ No

If Yes, for how long: …………………………..

Exposure to sunlight:

- Daily ☐
- 3-4 times weekly ☐
- 1-2 times weekly ☐
- Rarely ☐

Time outside home exposed to sunlight

- Less than one hour ☐
- 1-2 hours ☐

Are you suffering from?

Diabetes mellitus ☐ Yes ☐ No

Hypertension ☐ Yes ☐ No

Hypercholesterolemia ☐ Yes ☐ No

Heart diseases ☐ Yes ☐ No

Anthropometric measurements:

Weight: ………..Kg

Height: …………CM

Biochemical results:

Blood sugar level: ………………. mmol/L

Cholesterol: …………. mmol/L

25-hydroxyvitamin D: ……..nmol/L
